# Characterization of Hydrothermal Deposition of Copper Oxide Nanoleaves on Never-Dried Bacterial Cellulose

**DOI:** 10.3390/polym11111762

**Published:** 2019-10-27

**Authors:** W. Ross Warren, Dennis R. LaJeunesse

**Affiliations:** Department of Nanoscience, Joint School of Nanoscience and Nanoengineering, University of North Carolina Greensboro, Greensboro, NC 27401, USA; wrwarren@uncg.edu

**Keywords:** bacterial cellulose, nanoparticle synthesis, nanocomposite, biomaterial

## Abstract

Bacterial cellulose (BC) has attracted a great deal of interest due to its green synthesis and biocompatibility. The nanoscale dimension of BC nanofibers generates an enormous surface area that enhances interactions with water and soluble components within aqueous solution. Recent work has demonstrated that BC is a versatile platform for the formation of metal/metal oxide nanocomposites. Copper oxide (CuO) is a useful material to compare nanomaterial deposition on BC with other cellulosic materials because of copper’s colorimetric reaction as it forms copper hydroxide (Cu(OH)_2_) and transitions to CuO. In this research, we found that never-dried BC readily deposits CuO into its matrix in a way that does not occur on cotton, dried BC, or regenerated cellulose fibers. We conclude that hydroxyl group availability does not adequately explain our results and that intrafibrillar pores in never-dried BC nanofibers play a critical role in CuO deposition.

## 1. Introduction

Cellulose is a linear chain polysaccharide that is composed of β(1→4) linked d-glucose subunits and is the most abundant biopolymer on earth [1]. Nanocellulose materials are a collection of nanomaterials that include nanofibrillated cellulose (NFC), nanocrystalline cellulose (NCC), and bacterial cellulose (BC) [2,3,4]. The nanoscale dimensions of cellulose in these materials generates an enormous surface area that enhances interactions with water and soluble components within aqueous solution [5]. Nanoscale cellulose has attracted a great deal of interest due to its biodegradability, mechanical properties, and sustainability [4,5]. BC is a highly pure and crystalline cellulosic material that is secreted from certain bacteria (i.e., *Gluconacetobacter hansenii*); each bacterium produces a single nanocellulose fiber which entangles together with other nearby fibers to create a gel-like non-woven mat called a pellicle [3,5,6]. Purified BC pellicles are transparent and durable materials that are composed of a nonwoven mat of cellulose ribbons; each BC nanoribbon is 40–60 nm wide, 10–20 nm tall, and has a crystal structure that is predominately cellulose Iα [7,8,9]. In applied nanocomposite materials, BC has been used for supercapacitors, ion detection, pH sensing, and glucose sensing [10,11,12]. 

Recently, BC has been a highly active area of research as a synthetic platform for the generation of metal and metal oxide nanocomposites. A recent and extremely comprehensive review has shown that BC functions as the foundation for BC-metal oxide nanocomposites as well as a range of BC-metal sulfide and BC-metal nanocomposites [13]. Both solvo- and hydro-thermal processes have been used to deposit a variety of metal oxide (e.g., ZnO, TiO_2_, and Fe_3_O_4_.) nanomaterials into the BC matrix [14,15,16,17]. In these examples, the BC nanocomposite is chemically stable with the nanomaterial incorporated throughout the BC matrix. More recent work describes a hydrothermal process for making a BC–CuO nanocomposite [18].

Although recent work has demonstrated that BC is an extremely versatile substrate for the generation of metal/metal oxide nanocomposite materials, little work has been done to describe the properties of BC that enable these synthetic reactions [13]. Much of the work that describes deposition of metal/metal oxide nanomaterials into BC matrixes suggests that there is an interaction between the solubilized metal ion and the hydroxyl groups of cellulose which facilitates nanomaterial deposition. In this paper, we use CuO as a model nanomaterial to compare nanomaterial deposition on BC with other cellulosic materials due to its distinct XRD spectrum and colorimetric products. Furthermore, CuO and other copper materials have significant roles in many catalytic, energy, and semiconductor applications [19,20]. In these experiments we found that BC mediated deposition of CuO resulted in the formation of CuO nanoleaves under reaction condition that were far milder and more environmentally friendly than other CuO nanoleaf syntheses [18,21]. These results suggest that never-dried BC is uniquely suited as a nanoscale scaffold or platform for nanomaterial deposition when compared to other cellulosic materials.

## 2. Methods

### 2.1. Materials

Yeast Extract, d-mannitol, and Citric Acid were purchased from Sigma-Aldrich (Sigma-Aldrich, St. Louis, MO, United States). Anhydrous Copper Sulfate, NaOH pellets, Na_2_HPO_4_, Peptone, and 28% Ammonia were purchased from Fisher Scientific (Thermo Fisher Scientific, Waltham, MA, USA). Pellicles of BC were collected from cultures of Gluconacetobacter hansenii (ATCC 23769, American Type Culture Collection, Manassas, VA, USA).

### 2.2. Bacterial Cellulose Production

The Gluconacetobacter hansenii bacteria were cultured in media with the following composition: 2% (*w/v*) d-mannitol, 0.5% (*w/v*) Yeast extract, 0.5% (*w/v*) Peptone, 0.27% (*w/v*) Na_2_HPO_4_, and 0.125% (*w/v*) Citric Acid. Bacteria were cultured in 20 mL of media in 85 mm × 15 mm, or 100 mm × 15 mm, in petri dishes for 4 days at 30 °C. After 4 days, pellicles were processed in 0.1M NaOH at ~95 °C for 1 h to remove all bacteria and bacterial biofilm materials other than the BC matrix. Pellicles were then washed with reverse osmosis (RO) water until a neutral pH was obtained. Pellicles were either used immediately or stored in RO water at room temperature. Pellicles in storage were boiled in 0.1 M NaOH for 30 min and rinsed with RO water until at a neutral pH immediately prior to use.

### 2.3. Characterization of BC/Metal Oxide Nanocomposites

The FTIR spectra were collected on an Agilent 670 FTIR Spectrometer (Agilent, Santa Clara, CA, USA) in attenuated total reflectance (ATR) mode. BC pellicles were air-dried and folded before FTIR analysis. Morphology of nanoparticles was analyzed via scanning electron microscopy. The scanning electron micrographs were obtained using a Zeiss Auriga FIB/FESEM with EDX (Zeiss, Jena, Germany). Scale bars were added using ImageJ software (U.S. National Institutes of Health, Bethesda, Maryland, USA). To measure the progress of Cu(OH)_2_ formation, we performed UV–Vis spectral analysis on an Agilent 6000i UV–Vis Spectrophotometer (Agilent, Santa Clara, CA, USA) with a spectrum obtained every 2 min.

### 2.4. XRD Analysis

**Figure 1:**
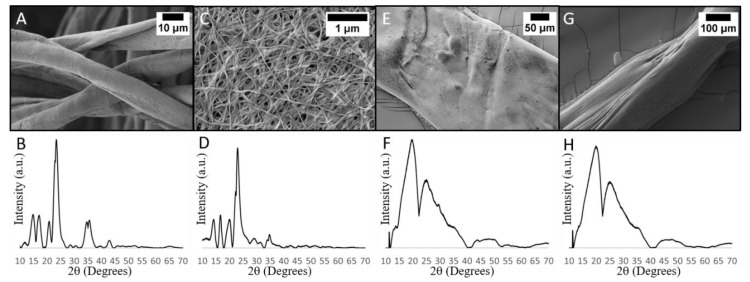
CuO deposition process performed on plain cotton, dried BC, and regenerated cellulose fibers: SEM images (top) and XRD spectra (bottom) are shown for plain cotton (**A**,**B**), dried BC (**C**,**D**), regenerated cotton (**E**,**F**), and regenerated never-dried BC (**G**,**H**).

The crystallinity of BC samples with and without metal oxide deposition were characterized using a Rigaku (Agilent) Gemini XRD (Rigaku, Tokyo, Japan). The samples were air-dried and then ground with a mortar and pestle. The powder was loaded into a borosilicate tube for analysis. For all XRD spectra, other than Figure 1B,C, a copper source was used at 40 V and 40 mA. 

For Figure 1F,H, a Molybdenum source at 40 V and 40 mA was used. The 2θ values of these spectra were then converted via d spacing to Copper 2theta values by the following process. The equation d = λ/(2.0 × sin(0.5 × 2θ × D2R)) was obtained from a U.S. Geological Survey website; with the wavelength of the x-rays λ = 1.5418Å which is a weighted average of Copper’s Kα1 and Kα2, and D2R = 0.0174532925199433 for converting between degrees and radians [22]. The above equation was solved for 2θ to give the following equation.

$$2\theta =\frac{\sin^{-1}\left(\frac{\lambda }{2d}\right)}{0.5*D2R}$$

The above equation was used to calculate a new 2θ value for each point on the Molybdenum spectra using d-values from the raw data file. To validate this method, a previously analyzed BC–CuO composite sample was analyzed via this process and the results were compared to a previously generated Copper source spectrum. The CuO and BC peak locations from the shifted spectrum agreed with peaks from the original Copper spectrum (Figure S4).

### 2.5. Copper(II)Oxide Composite Formation 

Pellicles were soaked in 50 mM CuSO_4_ for 1 h and then rinsed with RO water 3–5 times. Rinsed pellicles were then added to 0.1 M NaOH for 5 min and then transferred to 0.1 M NaOH at ~60 °C for 30 min. The BC–CuO composite was rinsed until at a neutral pH and stored in water or dried for analysis.

### 2.6. Regenerated Cellulose Fibers

The following procedure is a modified protocol from a video on the NileRed Youtube channel which demonstrates how to dissolve and regenerate cellulose using Schweizer’s reagent as the solvent [23,24,25,26].

To make the Schweizer’s reagent, 100 mL of a 50 mM CuSO_4_ solution was made by dissolving anhydrous CuSO_4_ in dH_2_O. 25 mL of 0.4 M NaOH was added to the CuSO_4_ solution to form Cu(OH)_2_. The solution was stirred and kept on ice until Cu(OH)_2_ formed. Moisture was removed from the Cu(OH)_2_ via vacuum filtration until a thick paste formed. The Cu(OH)_2_ paste was transferred to a graduated cylinder and 10 mL of 28% ammonia was added. The solution was stirred until the Cu(OH)_2_ dissolved completely and the solution took on a deep purple color. 

Once the Schweizer’s reagent was made, excess water was removed from never-dried BC, to prevent diluting the solution, by blotting on filter paper. Both cotton and never-dried BC were dissolved in batches until saturation and undissolved material was then removed. The now viscous solution was drawn into a luer-lok syringe, a 23G needle was attached, and the solution was ejected into a 10% sulfuric acid solution. For both never-dried BC and cotton, the regenerated fibers were formed by dissolution in Schweizer’s reagent and subsequent injection into 10% sulfuric acid [23,24,25,26]. The regenerated fibers were stirred in the 10% sulfuric acid until their blue color transitioned to opaque white. The regenerated fibers were rinsed until at a neutral pH and stored in dH_2_O.

## 3. Results and Discussion

### 3.1. CuO Nanoleaf Synthesis in a BC Matrix

Before we can compare BC–CuO synthesis to other cellulosic materials, we must first characterize our BC–CuO nanocomposite. Typically, the first step in CuO synthesis involves the generation of Cu(OH)_2_ through the addition of NaOH to an aqueous solution that contains a copper salt and ammonia. Cu(OH)_2_ is a blue insoluble precipitate that, when heated, will transform via a dehydration and recrystallization reaction into a brown CuO precipitate [20,27]. The particle size, morphology, and crystallinity of the CuO nanomaterial is determined by reaction conditions and reagents [27,28,29,30]. For instance, the addition of increasing ammonia caused a transition from nanoplate to nanowire CuO particles [31]; while the addition of other amines to the reaction yields belts or leaves composed of CuO [32].

**Figure 2:**
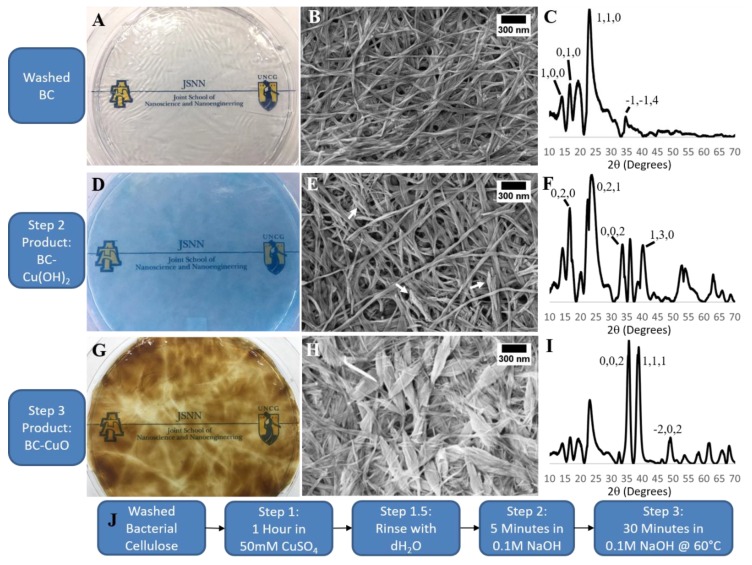
BC, BC–Cu(OH)_2_, and BC–CuO Nanoleaf Composite: (**A**–**C**) Plain BC; (**A**) purified, never-dried BC pellicle demonstrating clear, colorless material, (**B**) SEM of a BC pellicle, (**C**) XRD of a BC pellicle; cellulose Iα peaks indicated. (**D**–**F**) In-situ formation of Cu(OH)_2_ in BC matrix; (**D**) never-dried BC pellicle after Cu(OH)_2_ deposition, (**E**) SEM of the BC–Cu(OH)_2_ nanocomposite with spikes of Cu(OH)_2_ indicated (arrows), (**F**) XRD of BC–Cu(OH)_2_ nanocomposite; Cu(OH)_2_ peaks indicated. (**G**-**I**) BC–CuO nanoleaf composite; (**G**) never-dried BC–CuO pellicle, (**H**) SEM of BC–CuO nanoleaf composite, (**I**) XRD of BC–CuO nanoleaf composite; CuO peaks indicated; (**J**) Outline of the CuO deposition process we performed.

We adapted the common solution-based synthesis of CuO for the deposition of CuO onto the BC platform; however, this reaction only involves CuSO_4_, NaOH, and a purified BC without additional capping and/or stabilizing components. In the first step of the in-situ BC mediated CuO deposition process; we incubated purified pellicles in 50 mM CuSO_4_ for 1 h and then removed excess CuSO_4_ through a series of extensive rinses with RO water. We then incubated the Cu^2+^ saturated BC pellicle in 0.1 M NaOH, for 5 min, which resulted in the immediate in-situ deposition of crystalline Cu(OH)_2_ within the BC matrix as indicated by XRD and the pellicle taking on a characteristic deep blue color (Figure 2D,F) [19]. We monitored the colorimetric reaction of the BC pellicle via time-lapse UV–Vis spectroscopy and observed an absorption peak at 640 nm after 4–6 min post 0.1 M NaOH treatment (Supplemental Figure S1). SEM micrographs confirmed the deposition of nanomaterial with the presence of spiky depositions within the BC matrix (Figure 2E arrows). In the final step of this process, we heated the BC–Cu(OH)_2_ pellicle to 60 °C in 0.1 M NaOH for 30 min. This resulted in the transformation of the Cu(OH)_2_ to CuO, via a dehydration and recrystallization reaction, characterized by the BC pellicle turning brown (Figure 2G) [28]. SEM showed that the CuO had a nanoleaf morphology (Figure 2H) [33]. XRD analysis of the BC–CuO material confirmed that the nanoleaves are indeed crystalline CuO particles as the 2θ peaks at 35.5° and 38.5° match the [0,0,2] and [1,1,1] crystal faces of CuO, respectively (Figure 2I) [19]. The deposition of CuO onto the BC matrix was done under conditions that were faster, had a lower temperature, and without the use of capping reagents [20,27].

### 3.2. Comparison of Never-Dried BC and Other Cellulosic Materials after CuO Deposition Process

Recent research on BC nanocomposites has hypothesized that an interaction between metal ions and cellulose hydroxyl groups is responsible for nanomaterial deposition on BC [11,13,18]. To our knowledge, there has not been a study which further investigates this hypothesis. Since BC is chemically identical to other cellulosic materials, this hypothesis can be tested by performing the CuO deposition process shown in Figure 2 on other cellulosic materials. If the CuO deposition on never-dried BC is indeed facilitated by the adsorption of Cu^2+^ ions on cellulose hydroxyl groups, then we would also expect cotton and dried BC to facilitate CuO deposition. However, when we performed the CuO deposition process from Figure 2 on cotton, and dried BC, we observed no CuO deposition on either material (Figure 1A–D).

The difference between never-dried BC and the cellulosic materials in Figure 1A–D is that never-dried BC has never been dehydrated. A whole never-dried BC pellicle has a thickness of a few millimeters and is comprised of ~99% water. Upon drying, the pellicle undergoes an irreversible gel-to-film transition in which it shrinks to a thickness of less than 10 µm [7,34]. Since cellulose readily dries out when exposed to air, the BC must be stored in water to retain its never-dried state. 

Furthermore, in a process which has parallels to BC’s gel-to-film transition, cellulose in the form of kraft wood pulp undergoes a largely irreversible loss of water in a process called hornification. Briefly, hornification is a process in which pores close and cellulose fibers bond with each other when no longer separated by water. This process takes place both within and between cellulose fibers as the individual cellulose polymers bond with each other during pore closure. This process affects the hydroxyl groups of cellulose as hydrogen bonds are thought to play a major role in hornification [35,36]. One possible explanation for the lack of deposition on dried BC and cotton is that the hydroxyl groups are simply not accessible to Cu^2+^ and SO_4_^2-^ ions due to inter- and intramolecular hydrogen bonding which occurs during drying.

Regenerated cotton fibers formed by Schweizer’s reagent (i.e., cuprammonium rayon) have been shown to possess a large number of accessible hydroxyl groups [37]. If accessible hydroxyl groups in never-dried BC facilitate CuO deposition, then regenerated cotton should show CuO deposition since it has accessible hydroxyl groups. However, when we perform the CuO deposition process, outlined in Figure 2, on regenerated cotton fibers we see the fibers have the expected cellulose II crystal structure but fail to find any CuO deposition (Figure 1E,F). The lack of CuO deposition on regenerated cotton fibers suggests that never-dried BC’s CuO deposition is not solely facilitated by accessible hydroxyl groups.

### 3.3. Bacterial Cellulose Fiber Organization as a Mechanism for CuSO_4_ Retention during Rinsing

The lack of CuO deposition in on rinsed regenerated cotton fibers (Figure 1E,F) warrants further discussion of cellulose structure in never-dried BC. Each BC nanofiber is a collection of smaller crystalline cellulose fibers. The smaller fibers are extruded from bacterial pores, then aggregated, and bond together in the extracellular space [38,39]. The smaller fibers do not completely bond as evidenced by neutron scattering research that showed never-dried BC nanofibers are nanoporous fibers through which water can diffuse [40]. 

As previously mentioned, when we subjected regenerated cotton fibers to our CuO deposition process, we did not observe CuO deposition; however, when the regenerated cotton was not rinsed after being soaked in CuSO_4_ we did observe significant CuO deposition (Figure S2). This result is similar to previous research which proposed that intrafibrillar pores in regenerated cellulose fibers can serve as spaces for Fe_2_O_3_ formation [41]. The key difference between performing CuO deposition on regenerated cotton and never-dried BC is that rinsing removes most, if not all, adsorbed Cu^2+^ and SO_4_^2-^ ions from regenerated cotton’s intrafibrillar pores. Conversely, never-dried BC nanofibers retain some Cu^2+^ and SO_4_^2-^ ions after being rinsed, which can then be reacted to deposit CuO.

Given that both never-dried BC and regenerated cotton fibers are fibers with intrafibrillar pores, the biggest difference between them is the fiber diameter. Dehydrated BC nanofibers are ~40 nm in diameter while our regenerated cotton fibers have a diameter of at least 50 µm when dehydrated. It is also important to note that the regenerated cotton fibers were dried before SEM imaging, and the fibers were significantly larger prior to drying. The massive difference in diameter suggests that the intrafibrillar pores of our regenerated cotton fibers are much larger than those of never-dried BC nanofibers. We hypothesize that during rinsing, due to never-dried BC’s much smaller intrafibrillar pore size relative to regenerated cotton, water only removes Cu^2+^ and SO_4_^2-^ ions from the interfibrillar pore space and does not penetrate never-dried BC’s intrafibrillar nanopores. This process leaves behind Cu^2+^ and SO_4_^2-^ ions in the intrafibrillar pore space of never-dried BC which then deposits as CuO.

If our intrafibrillar pore size hypothesis is correct, we would expect that fibers made of regenerated never-dried BC would behave the same as regenerated cotton since the nanopores in never-dried BC would be destroyed during dissolution. We employed the previously mentioned Schweizer’s reagent process to make regenerated cellulose fibers from never-dried BC. These regenerated fibers were not dried and kept in water prior to testing. When we performed the CuO deposition process, outlined in Figure 2, on regenerated fibers made from never-dried BC, we saw no CuO deposition (Figure 1G,H). This result demonstrates that the retention of Cu^2+^ and SO_4_^2-^ ions during rinsing is not simply a property intrinsic to never-dried BC and reinforces our hypothesis that fiber organization is responsible for the different responses displayed by the cellulosic materials we examined.

### 3.4. When is Never-Dried BC Needed during BC–CuO Synthesis?

**Figure 3:**
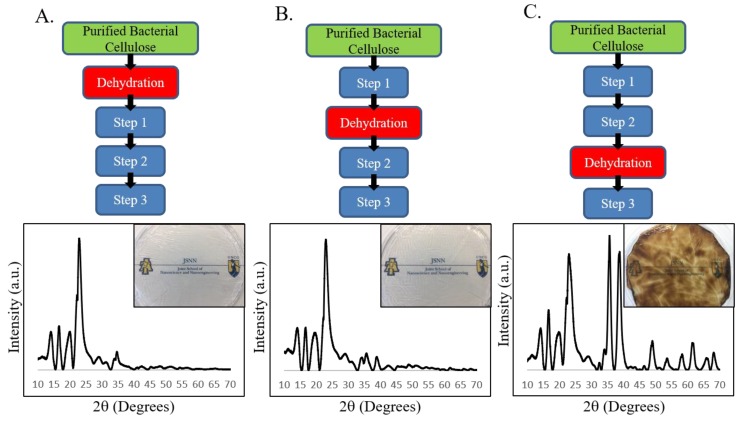
Hydration state of BC is essential for formation of CuO nanomaterial: Step 1, incubation of purified BC pellicle in 50 mM CuSO_4_; Step 2, formation of BC–Cu(OH)_2_ composite material via incubation of the rinsed copper treated BC pellicle in 0.1 M NaOH; Step 3, heating BC–Cu(OH)_2_ composite at 60 °C for 30 min. (**A**) Dehydration of BC pellicle prior to Step 1 results in no formation of CuO material as evidenced by the XRD spectrum lacking the CuO signature and a pellicle with no color change (inset); (**B**) Dehydration of BC pellicle after copper treatment (Step 1) but before the formation of Cu(OH)_2_ (Step 2) also results in the failure to form CuO as evidenced by XRD and a lack of color change in the pellicle (inset); (**C**) Dehydration of the BC pellicle after Cu(OH)_2_ formation (Step 2) and prior to the heating (Step 3) results in the deposition of CuO material in the BC matrix, as evidenced by XRD and a brown colorimetric change to the pellicle (inset).

To further examine the role of intrafibrillar nanopores during CuO deposition we performed the in-situ BC mediated CuO deposition, shown in Figure 2, on BC pellicles dried before different steps in the process. By drying out the BC before each step in the CuO deposition process we can gain insight into the role played by BC’s intrafibrillar nanopores because the pores close when BC is dried. The results of this experiment are shown below in Figure 3.

When the pellicle was dried prior to CuSO_4_ incubation (dried prior to Step 1, Figure 3A) the BC was in the same dehydrated state as cotton. Performing the CuO deposition process on dried BC resulted in no colorimetric change of the pellicle and no CuO XRD signature. This is the same result shown in Figure 1C,D. Drying after CuSO_4_ incubation and prior to NaOH incubation (dried prior to Step 2) resulted in very little CuO deposition. This is indicated by the lack of any colorimetric change in the pellicle and the lack of a strong CuO XRD signature (Figure 3B). This result is not surprising when viewed in the context of intrafibrillar pore closure. If intrafibrillar pores, which close during drying, contain Cu^2+^ and SO_4_^2-^ ions, then we would expect the ions to be expelled when the pores close. The expelled Cu^2+^ and SO_4_^2-^ ions then recombine and form the same CuSO_4_ hydrate that forms when the water in a CuSO_4_ solution is allowed to evaporate (Figure S3). The water soluble CuSO_4_ Hydrate is removed during subsequent rinsing which results in very little CuO deposition. Finally, when the pellicle was not dried until after step 2 (dried prior to Step 3, Figure 3C), we observed a colorimetric change and XRD signature which closely resembles the BC–CuO nanocomposite shown in the bottom row of Figure 2.

Based on the results of Figure 3, the association between Cu(OH)_2_ and the BC nanofibers is preserved during drying and subsequent rinsing which allows CuO to form during Step 3 of the process. However, an association between Cu^2+^ ions and BC nanofibers was not established when BC is dried before soaking in CuSO_4_ and is not preserved when BC is dried after soaking. In this procedure, the Cu(OH)_2_, which forms during Step 2, is only deposited if an association between Cu^2+^ ions and BC nanofibers is established during Step 1 and maintained during rinsing between Steps 1 and 2 (Step 1.5). This result further supports our hypothesis that the intrafibrillar nanopores of never-dried BC facilitates the retention of Cu^2+^ ions during rinsing between steps 1 and 2 of the CuO deposition process. It is this feature that differentiates never-dried BC from other cellulosic materials, and we hypothesize that it is due to the nanoscale size of its intrafibrillar pores.

### 3.5. CuO Integration into the BC Matrix

**Figure 4:**
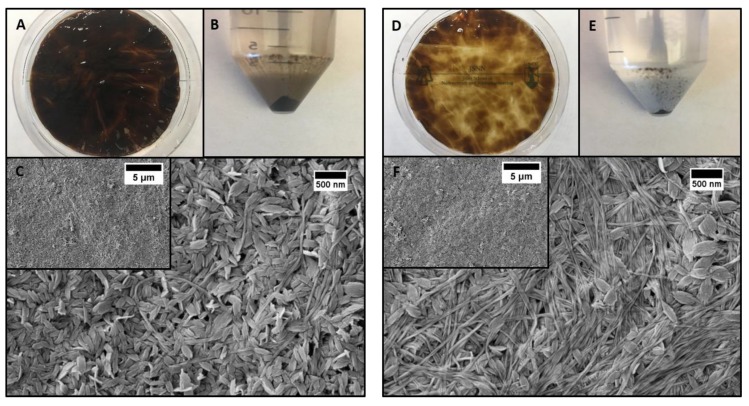
Removal of non-adsorbed CuSO_4_ via thorough rinsing: (**A**–**C**) BC–CuO with no rinse after 1 h in 50 mM CuSO_4_ (no rinse after step 1); (**A**) Picture of BC–CuO, (**B**) Picture of pellet after centrifugation of step 3 reaction solution at ~1750 RCF for 10 min, (**C**) SEM of BC–CuO. (**D**–**F**) BC–CuO with thorough rinsing after 1 h in 50 mM CuSO_4_ (with rinse after step 1); (**D**) Picture of BC–CuO, (**E**) Picture of pellet after centrifugation of step 3 reaction solution at ~1750 RCF for 10 min, (**F**) SEM of BC–CuO.

Unlike solution phase CuO nanomaterial synthesis, the CuO nanoleaves generated by this method were stably integrated in the BC matrix and resist removal even with significant agitation. Also, when never-dried BC pellicles were not thoroughly rinsed after soaking in CuSO_4_ a greater overall amount of CuO was deposited (Figure 4C). Without thorough rinsing, much more CuO was not stably integrated in the BC matrix and precipitated into solution. This is apparent when comparing the amount of free CuO precipitated by an unrinsed pellicle (Figure 4B) to that of a thoroughly rinsed pellicle (Figure 4E). This result further reinforces that there are at least two levels of arrangement of the Cu^2+^ ions within the BC matrix: one in which the Cu^2+^ ions are stability associated with the BC nanofibers and a bulk phase in which the Cu^2+^ ions are not associated with the BC nanofibers.

The deposition of CuO is clearly non-uniform on the scale of an entire BC pellicle (Figure 4A,D). This brings up questions as to how non-uniform nanomaterial deposition could ultimately affect material performance. One cause of non-uniform deposition is how the BC is handled during processing. In our experiments, the Cu^2+^ loaded BC pellicles were removed from solution by a handheld sieve. This sieve caused the Cu^2+^ loaded pellicle to become folded and this could cause BC to expel Cu^2+^ ions at the creases. Another potential cause of non-uniformity is the BC itself. BC pellicles are not uniform materials because they are generated by free floating bacteria in media. This will inevitably lead to a non-uniform BC density across the pellicle on the nanoscale. However, depending on the application, other properties such as weight percentage could be more important than uniformity (i.e., photocatalysis). Furthermore, the ability of never-dried BC to retain some adsorbed CuSO_4_ after rinsing allows for further control over bulk nanocomposite properties. These concerns are highly application specific, and research will be needed to tailor BC nanocomposite synthesis to each application’s needs.

## 4. Conclusions

In this paper, we demonstrated that BC facilitates the formation of CuO nanoleaves that are intimately integrated into the mechanically stable BC nanofiber matrix. The deposition reaction takes place under lower temperature conditions and with environmentally friendly reagents. We also provide a detailed characterization of CuO nanoleaf deposition into the BC matrix. In this paper we show that having accessible, hydrated, hydroxyl groups is not enough to explain why we observed CuO deposition on never-dried BC and not on the other cellulosic materials we tested. We propose that the primary feature of never-dried BC that facilitates CuO deposition is the intrafibrillar nanopores in each BC nanofiber. The exact interaction of solutes with the nanopores and how processing methods affect these interactions are open areas of future research. These details will be critical for the large-scale production of CuO and other metal oxide materials that use BC or other cellulosic materials as the deposition substrate. Furthermore, other crystalline polymeric materials may facilitate similar reactions, and further understanding of never-dried BC will aid the identification of these polymers.

## Supplementary Materials

The following are available online at https://www.mdpi.com/2073-4360/11/11/1762/s1, Figure S1: Time-lapse UV–Vis spectrum, Supplemental Figure S2: SEM Images of Regenerated Cotton Fibers with CuO deposition, Supplemental Figure S3: XRD characterization of Step 1 product after drying, Supplemental Figure S4: Demonstration of Molybdenum source 2-theta shift with BC–CuO composite.
