# Supplementary material for: Characterization of Hydrothermal Deposition of Copper Oxide Nanoleaves on Never-Dried Bacterial Cellulose
Source: Polymers (Basel). 2019 Oct 27;11(11):1762. doi: 10.3390/polym11111762 (PMC6918234; doi:10.3390/polym11111762)
Supplement: Supplementary file 1 [file polymers-11-01762-s001.pdf]

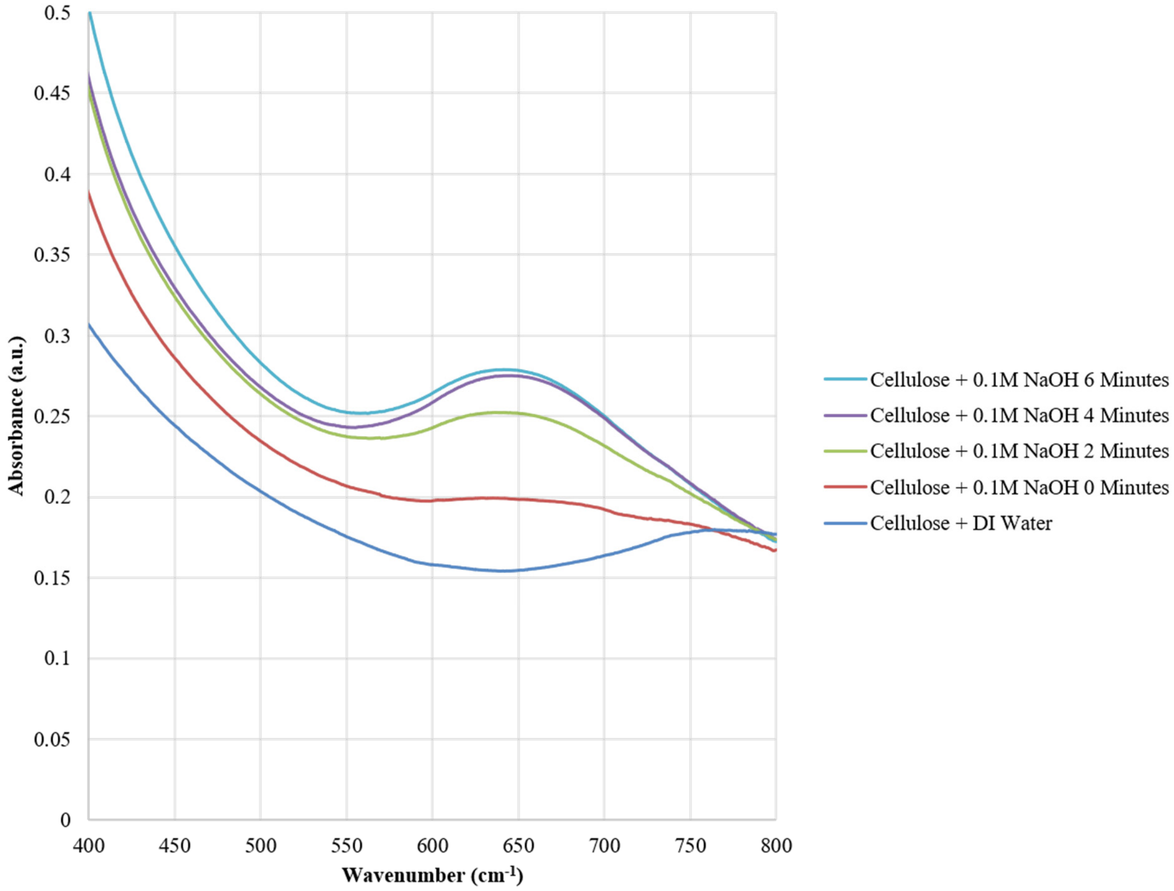

Supplemental Figure S1. Time-lapse UV-Vis spectrum. Bacterial nanocellulose was soaked in 50mM $CuSO_4$ for 1 hour, rinsed, and placed a UV-Vis cuvette with 0.1M NaOH. Scans were taken every 2 minutes. Blue, purified and untreated BC pellicle; Red, time 0 after NaOH treatment.

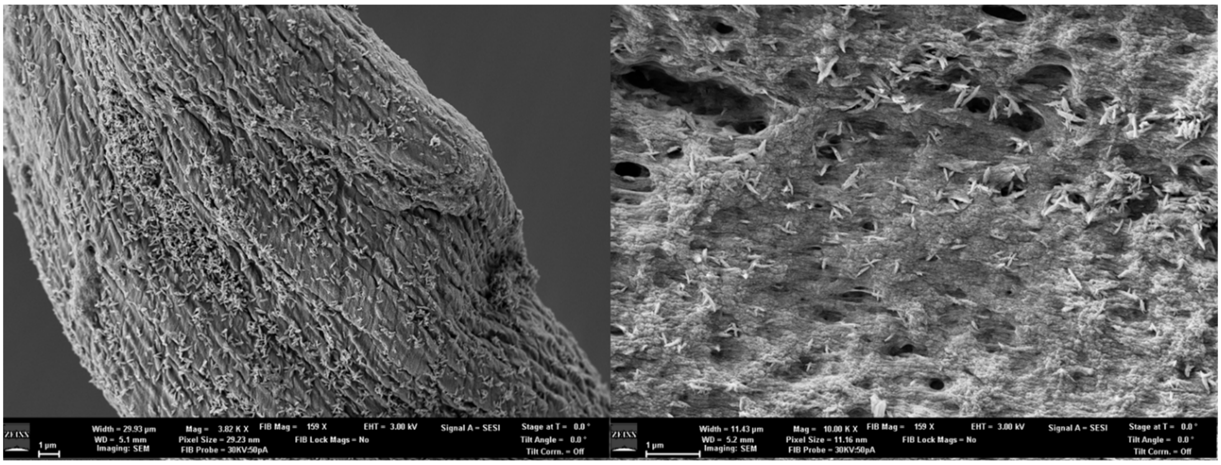

Supplemental Figure S2. SEM Images of Regenerated Cotton Fibers with CuO Deposition.

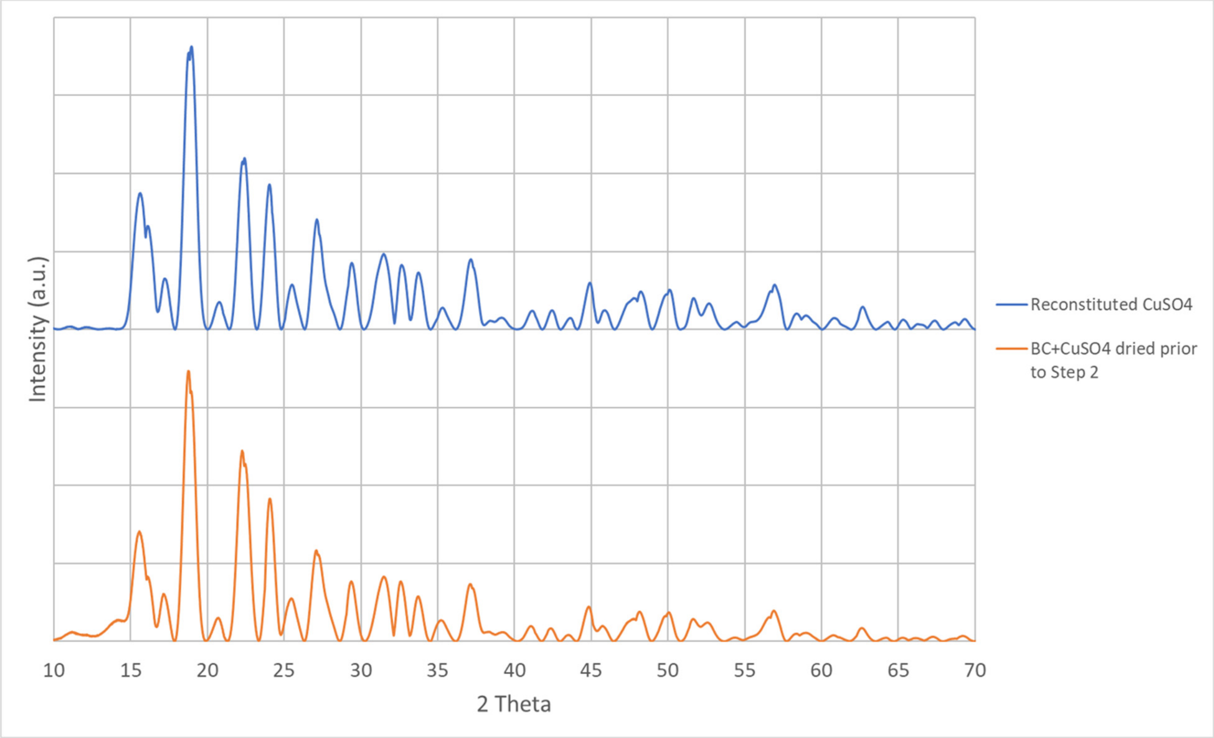

Supplemental Figure S3. Characterization of Step 1 product after drying. The bottom XRD spectrum is from the product formed by drying a pellicle after soaking in 50mM $CuSO_4$ for 1 hour without subsequent rinsing. The top spectrum is from an aliquot of 50mM $CuSO_4$ from which the water was allowed to evaporate. The spectra indicate that the same $CuSO_4$ hydrate formed under both conditions.

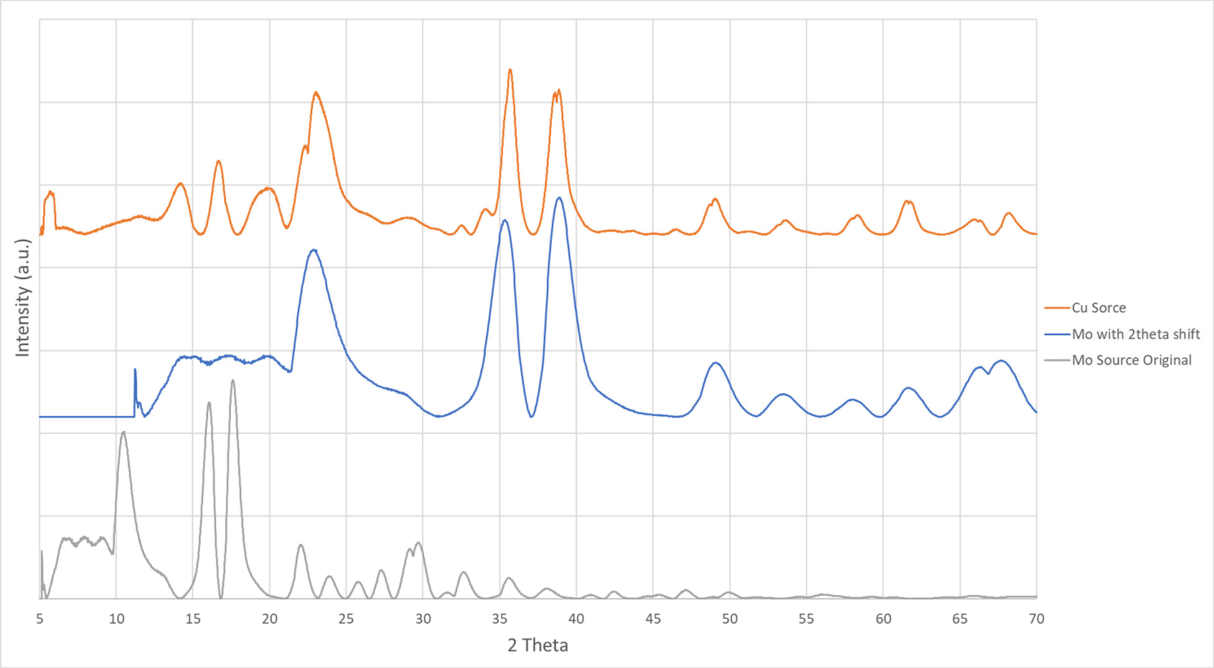

Supplemental Figure S4. Demonstration of Molybdenum source 2theta shift with BC-CuO composite. (Bottom) Original Molybdenum XRD spectrum, (Middle) Molybdenum spectra with shifted 2theta, (Top) BC-CuO sample with spectrum from Copper source.
